# Scoring Microbiota Function: A Proposal to Use Features of Evolutionary, Symbiotic Innovation to Recognize a “Healthy” Human Gut Microbiota

**DOI:** 10.1080/29933935.2024.2376543

**Published:** 2024-07-17

**Authors:** Gerald W. Tannock

**Affiliations:** Department of Microbiology and Immunology, University of Otago, Dunedin, New Zealand

**Keywords:** Gut, microbiota, function, health, symbiotic, dysbiosis, evolution

## Abstract

Research concerning the significance of the bacterial community of the human colon (gut microbiota or microbiome) in the etiology of diseases has depended in large part on molecular and bioinformatic tools to assemble catalogs of bacterial diversity. This article proposes that the gut microbiotas of humans are collectively a metacommunity whose functions are characteristic and consistent across all healthy humans. The pathway of evolutionary innovation in the development of the symbiosis between humans and gut microbiotas is known. Therefore, it is suggested that functional scoring of these long-lasting symbiotic innovations will reap greater benefits in delineating health or disease than can comparative taxonomic analysis. Adoption of a function-scoring approach would offer opportunities for emerging researchers, worldwide, to form multidisciplinary teams to develop essential methodologies to advance this gut microbiota research.

## Introduction

The human colon contains a microbial community composed mostly (99.9%) of bacterial species.^1^ Archaea and fungi are detectable as small proportions of the microbiota.^2,3^ Viruses are present, mainly as temperate bacteriophages of bacteria.^4,5^ The taxonomic composition of the community, usually described as the gut microbiota or microbiome, has been studied using high throughput DNA sequencing methods coupled with sequence data analysis using complex bioinformatic approaches including artificial intelligence (machine learning).^1,6,7^ The main goal of this research has been to define the “healthy human microbiota” in terms of the proportions (relative abundances) of taxa comprising the community. The hope has been that aberrant (dysbiotic) communities can be recognized as causative factors of some human diseases and conditions.^8^ However, it is increasingly apparent from the results of these taxon-centric studies that it is impossible to define a healthy or normal gut microbiota (microbiome) because of the large variation in bacterial diversity between individual human gut microbiotas.^9–11^ Indeed, some would consider that an over-enthusiasm for taxonomic investigations has led to overinterpretation of the significance of “the microbiome”.^12^

In ecological terms, it would be better to view the disparate gut microbiotas of individual humans collectively as comprising a “metacommunity” which has characteristic functional features (outputs) despite taxonomic variation.^13,14^ Studies that focus on the evolutionary co-divergence of *Homo sapiens* and the gut microbiota reveal that the composition and function of the metacommunity has been greatly influenced by the human dependence on cereals as dietary staples that persists even in industrialized societies.^15^ Unlike herbivorous animals, humans have little capacity to store dietary fiber for microbial fermentation but have huge ability to rapidly digest starch, animal protein and lipid in the proximal digestive tract. Nevertheless, forms of starch resistant to human digestion as well as structural plant components (hemicelluloses) pass to the colon where about 80% is degraded and fermented by bacterial species in a metabolically integrated system.^16–18^ Critical digestion capacities of the primary degraders in the community are encoded largely by genetic loci known as Polysaccharide Utilisation Loci (PULs).^19^ Recognition of these genetic features from genome-sequencing studies supports the concept of a long-standing, special relationship between human diet, cereal-derived polysaccharides, and the gut microbiota. Gnotobiotic mouse experiments with gut microbes from humans show that ability to form populations in the gut is associated with ability of bacteria to utilize carbohydrates from food (plant glycans) or host sources (mucins, unconjugated bile acids) for growth.^20^ This gnotobiotic experimentation emphasizes the foregoing view that catabolism of dietary and host-derived substrates by the microbiota is a key feature of the symbiosis between gut microbiota and the host.

**Figure 1.**
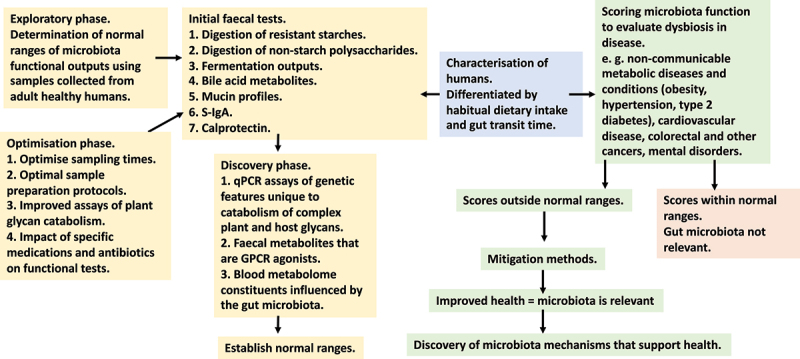
A framework for developing functional scoring of the gut microbiota to detect dysbiotic associations in human diseases.

Functional redundancy in the bacterial world results in communities of different taxonomic composition that have the same emergent properties.^21^ Thus, more likely to succeed in differentiating between healthy and dysbiotic relationships would be an investigative approach by which the functioning of microbiotas was tested. Microbiota functions of well people could be tested first to determine the range of “normal values” pertaining to each of the tests, analogous to the use of “lab tests” of peripheral blood, widely used in medical diagnostics to assess functioning of the human body. To initiate discussion of this approach and provide “first steps” in future research, functions that might be used to assess the health of the symbiosis between gut microbiota and human host will be discussed. The overall developmental framework is provided in Figure 1.

The fermentation products resulting from the microbial catabolism of substrates in the gut are mainly short chain fatty acids (SCFAs; mostly acetate, propionate, butyrate in the ratio 3:1:1) as well as gases (hydrogen, carbon dioxide, methane), minor amounts of branched-chain fatty acids (isobutyrate, isovalerate) derived from fermentation of amino acids in the distal colon, a minor amount of the SCFA valerate, and pungent volatiles such as indoles (derived from tryptophan), hydrogen sulfide, and thiols (derived from sulfur-containing amino acids such as methionine).^22,23^ Of these metabolites, SCFAs are currently the most promising candidates for scoring because there is a substantial databank of quantitative information obtained by methodologies such as gas–liquid chromatography, ^1^H NMR spectroscopy, and ultrahigh-performance liquid chromatography-triple quadrupole mass spectrometry.^24,25^ Moreover, the SCFA profile detected in feces is known to vary qualitatively according to human physiology (colonic transit time), and quantitatively in pathologic states (inflammatory bowel diseases) and through marked differences in consumption of dietary fiber.^26–29^

Most of the bile acid molecules secreted in bile into the gut are salvaged by an enterohepatic circulation, but some molecules pass to the colon where they become substrates for bacterial metabolism.^30^ The major transformation carried out by bacteria is dehydroxylation of deconjugated bile acids to form secondary bile acids. About 80% of bile acids in feces of healthy people are secondary bile acids, but the proportion can be affected by disease. For example, a smaller proportion (about 50%), along with larger proportions of conjugated primary acids and sulfated bile acids, is detected in the feces of inflammatory bowel disease patients.^31^

Mucins (*O*-glycans; glycoprotein constituents of mucus that covers the epithelium of the gut) are catabolised by some members of the gut microbiota. Fecal profiles of mucin degradation products can be obtained by matrix-assisted laser desorption ionization time of flight mass spectrometry. It has been reported that fecal *O*-glycan profiles differ between healthy people and Crohn’s disease patients.^32^ This might reflect underlying genetic differences between patients and healthy humans but, at least, these results indicate that there is technology available to further investigate the diversity of profiles among humans in general.

The time taken for food to move through the gut varies between humans and is influenced by dietary composition and human physiology, including pathologies. Gut transit time affects temporal loading of nutrients in colonic regions which influences microbial ecology. Where colonic transit time is long, carbohydrates in the digesta are depleted and amino acids are used as an alternative energy source by the microbiota. Fermentation of amino acids results in branched chain SCFAs. Taxonomic composition of fecal microbiotas also changes. Longer transit time is reported to be associated with altered abundances of *Akkermansia muciniphila*, *Bacteroides*, *Alistipes* spp., *Subdoligranulum* sp., *Ruminococcus bromii*, and *Methanobacter smithii*.^33–35^ Gut transit time can be measured accurately as the duration of time from ingestion of blue dye within a standardized food to its first excretion of blue color within a stool.^36^ Use of this method shows that humans can be divided into three groups according to gut transit time: fast,<14 hours; normal 14–58 hours; slow, >59 hours. Finer temporal divisions may be useful. Fecal water content (usual range 54% to 87%) can be used as a surrogate measurement of stool consistency and has been considered to reflect gut transit time because slower passage through the colon provides more time for water absorption.^37,38^ These values are also influenced by the water-holding capacity of insoluble solids, and the water-absorbing ability of the participant.^39^ The Bristol Stool Form Scale is often used as a tool for subjective scoring of stool consistency but has only moderate correlation when used by untrained participants.^40^

About 80% of the immune-system cells of the body are associated with the digestive tract.^41^ This observation reflects the alimentary canal as a portal for the entry of food-associated antigens – and a potential portal for pathogens – into the body. Mechanisms are required to “tolerize” the body to immunostimulatory molecules in the diet, and to prevent the invasion of the mucosa and the systemic spread of pathogens. Additionally, the trillions of bacteria that make up the microbiota provide a diversity of antigens associated with bacterial cells and their extracellular products. Therefore, measuring nonspecific responses of immune cells associated with the gut to the presence of the microbiota could be useful. Much of the microbiota antigenic mass seems to be invisible to the sentinels of the immune system (for example, mucosal dendritic cells) which may require an “above-threshold” stimulus to react.^42^ The transport into the gut lumen of low-specificity secretory immunoglobulin A (S-IgA) by cells in the mucosa is part of the “invisible microbiota” phenomenon. About 24–74% of the bacterial cells in human feces are coated with S-IgA. Such cells are unable to enter the mucosa and are therefore restricted to a luminal existence.^43^ S-IgA can be measured in feces using enzyme-linked immunosorbent assay. More S-IgA is secreted in inflammatory bowel disease compared to in healthy people. Coating of bacteria can be measured by flow cytometry methods.^44^

Calprotectin is an oligomeric protein of molecular mass about 36.5 kDa that accounts for 60% of the cytosolic protein of neutrophils. It is resistant to intestinal and bacterial proteases and has a homogeneous distribution in feces. An elevated fecal calprotectin value indicates the migration of neutrophils to the intestinal mucosa which occurs during inflammation. Fifty milligrams of calprotectin per gram of feces is usually considered to be the upper limit of normal when measured using commercial kits commonly based on enzyme-linked immunosorbent assay.^45^

“Metabolomics” is the large-scale, non-targeted, quantitative analysis of low molecular weight metabolites detected within cells, body fluids, and tissues. Collectively, these small molecules and their interactions within a biological system constitute the “metabolome”. Some metabolites produced in the colon are absorbed and carried by the portal blood circulation to the liver where they are often chemically transformed before entering the systemic circulation where they can be studied in plasma/serum using metabolomics techniques (ultra-high-performance liquid chromatography linked to mass spectrometry). The results of recent investigations indicate that the activities of the gut microbiota have an influence on the plasma/serum metabolome.^46^ Of long-standing interest has been the link between blood levels of trimethylamine *N*-oxide (TMAO) and atherosclerotic cardiovascular disease.^47,48^ TMAO is the product of trimethylamine (TMA) oxidation in the liver and high and low TMAO producers can be detected. TMA can be produced from betaine, L-carnitine, or choline in the diet by the members of several taxonomic groups in the gut microbiota.^49^ Therefore, excitement was generated by the possibility that gut microbiota function could influence cardiovascular disease or, at least, provide a biomarker of disease. However, TMAO can be obtained directly from food, especially fish,^50^ and recent attempts to link plasma levels with cardiovascular disease have produced inconsistent results and suggest that renal excretion rates and diet are the most important factors influencing metabolomic results.^51^ Nevertheless, other microbial metabolites may be found in ongoing metabolomic studies that could be scored as indicators of health or disease, vastly expanding the number of functional evaluations.

Unabsorbed microbial metabolites produced in the colon might also provide useful features to score. Some are potential signaling molecules in mammalian physiology because they are agonists of G protein-coupled receptors (GPCRs). Some of these receptors are located in the intestinal epithelium and several bind small-molecule therapeutic agents, so they are of physiological significance.^52^ GPCR clone-libraries can be prepared and used in high throughput screens of bacterial metabolites produced in cultures of fecal bacteria.^52,53^ Although the selection of bacterial strains has so far been limited, bacterial metabolites that have the potential to modulate host physiology have been identified (for example, SCFAs, *N*-acyl amides [fatty acid compound mimicry], histamine, phenylalanine, tyramine, cadaverine, nicotinic acid).^54^ Studies with gnotobiotic mice confirm that in vitro (culture) observations have physiological relevance, at least to the extent that the metabolites are enriched in gnotobiotic gut relative to germfree.^52,54^ Further investigations might uncover candidate molecules for quantitative scoring in health and disease.

Variation in scores between healthy humans will be apparent due to individual factors such as genetics, environment, and age^55–57^ so there will be “normal ranges” just as there are for measurements of human physiology. Once these normal ranges are established for several symbiotic parameters, scores of microbiota function could be determined for people with well-defined diseases. In some cases, scores would lie outside normal values. An algorithm could be derived that would aid in assessing values and producing composite scores indicating malfunctioning symbiosis. This might be aided by artificial intelligence research. Where functional scores are abnormal, methods for restoration of microbiota function could be developed and, once normal scores were regained, effects on clinical status could be evaluated. Thus, relatively clear indications of the involvement of the microbiota in disease could be obtained through these intervention studies. Such translational studies might involve dietary modification, alteration of gut transit time, and/or administration of bacterial consortia with integrated metabolisms producing specific functional outputs, which might be developed in the future.

Molecular explanations of malfunction and restoration of normal function could follow using in vitro ecological experiments utilizing “synthetic” microbial communities to model specific functions occurring in the gut ecosystem.^58^ These model, in vitro communities could be investigated to generate regulatory information.^59–61^ Mutation of specific genes could test ecological fitness of bacteria to show the importance of particular bacterial attributes in underpinning symbiosis.^62^ Genetic loci encoding fitness determinants could then be measured as additional indicators of a normal microbiota using quantitative PCR screens of fecal microbiotas.

Admittedly, some limitations of a functional analytical approach can be perceived. The choice of human participants for developmental studies will be challenging because there is a need to consider human populations in “western”, “non-western”, “non-industrial”, “industrial”, and “transitional” situations. Industrialisation of societies tends to select for gut communities of lower diversity due to consumption of diets containing more refined grains, and fruit and vegetable cultivars that are less bulky and chewy.^63^ The fecal microbiotas of “non-industrial” people have greater relative abundances of *Prevotellaceae* and *Spirochaetaceae* compared to people in western countries. In contrast, “western” “industrial” diets select for *Bacteroides thetaiotaomicron* and *Akkermansia muciniphila*.^64^

The impact of medications used to treat diseases or conditions may affect the activities of the microbiota, and the impact of oral antibiotic treatment would need to be evaluated in preliminary studies.^65^ Temporal sampling of the microbiota should be carried out to reveal the dynamics of community functions. For example, how long after dietary intervention does modulation of the microbiota occur and is the effect transient or permanent? For some analyses, blended total 24-hour collection of feces may be necessary to reduce variability of values. More extensive and better assays of microbiota function (such as resistant starch digestion), together with better information about the structure of dietary plant glycans (many are poorly characterized) are required.^66^

Information about the diets of participants in gut microbiota studies has so far been rudimentary. This is a serious failure in the design of studies to date, considering the dependence of the microbiota on resistant starch and plant glycans in general as growth substrates. The importance of dietary analysis will have increased importance in functional studies because mitigation of abnormal function may require dietary improvement. It is important that nutritionists are included as members of research teams to develop this aspect of gut microbiota research. From a positive viewpoint, potential limitations are really opportunities for novel, world-wide research wherever suitable analytical equipment is available.

Overall, the development of methods for scoring gut microbiota function provides golden opportunities for multi-disciplinary teams composed of emerging researchers to develop new concepts and methodologies in gut microbiota research. These ecology-minded teams will move the emphasis from DNA sequencing and bacterial taxonomy to microbiota outputs. The human gut microbiota has been likened to an organ of the body;^67^ it is time to develop this concept further with emphasis on ecological function.

## Data Availability

Data sharing is not applicable to this article as no datasets were generated or analyzed during the current study.
